# The Structure of Working Memory and Its Relationship with Intelligence in Japanese Children

**DOI:** 10.3390/jintelligence11080167

**Published:** 2023-08-18

**Authors:** Yoshifumi Ikeda, Yosuke Kita, Yuhei Oi, Hideyuki Okuzumi, Silvia Lanfranchi, Francesca Pulina, Irene Cristina Mammarella, Katie Allen, David Giofrè

**Affiliations:** 1 Department of Special Needs Education, Joetsu University of Education, Niigata 943-8512, Japan; yosifumi@juen.ac.jp; 2 Department of Psychology, Faculty of Letters, Keio University, Tokyo 108-8345, Japan; 3 Cognitive Brain Research Unit (CBRU), Faculty of Medicine, University of Helsinki, 00014 Helsinki, Finland; 4 Faculty of Liberal Arts and Sciences, Chukyo University, Aichi 470-0393, Japan; 5 Faculty of Education, Tokyo Gakugei University, Tokyo 184-8501, Japan; 6 Department of Developmental and Social Psychology, University of Padua, 35131 Padova, Italy; 7 School of Education, University of Durham, Durham DH1 3LE, UK; 8 DISFOR, University of Genoa, 16121 Genova, Italy

**Keywords:** working memory, children, intelligence, short-term memory

## Abstract

There is a host of research on the structure of working memory (WM) and its relationship with intelligence in adults, but only a few studies have involved children. In this paper, several different WM models were tested on 170 Japanese school children (from 7 years and 5 months to 11 years and 6 months). Results showed that a model distinguishing between modalities (i.e., verbal and spatial WM) fitted the data well and was therefore selected. Notably, a bi-factor model distinguishing between modalities, but also including a common WM factor, presented with a very good fit, but was less parsimonious. Subsequently, we tested the predictive power of the verbal and spatial WM factors on fluid and crystallized intelligence. Results indicated that the shared contribution of WM explained the largest portion of variance of fluid intelligence, with verbal and spatial WM independently explaining a residual portion of the variance. Concerning crystallized intelligence, however, verbal WM explained the largest portion of the variance, with the joint contribution of verbal and spatial WM explaining the residual part. The distinction between verbal and spatial WM could be important in clinical settings (e.g., children with atypical development might struggle selectively on some WM components) and in school settings (e.g., verbal and spatial WM might be differently implicated in mathematical achievement).

## 1. Introduction

Working memory (WM) can be broadly defined as the capacity to actively maintain information for a short period of time (Baddeley 1986). The importance of WM relies on the fact that it can predict important outcomes, such as intelligence and academic achievement in children with both typical and atypical development (see Cornoldi and Giofrè 2014 for a review). Different models of WM have been proposed over the years (see Carretti et al. 2022 for a recent review). At the same time, the relationship between WM and intelligence has been extensively examined in adults, yet has scarcely been investigated in children (Gray et al. 2017). The aim of the current report was therefore to investigate the structure of WM and its relationship with intelligence.

### 1.1. The Structure of WM

Very early models, which were linked to memory processes in general rather than being focused on WM, introduced the concept of a single mechanism (i.e., short-term memory), which was distinguished from long-term memory, and was initially conceived as a single memory storage mechanism for maintaining information for a short period of time (Atkinson and Shiffrin 1968). This early formulation, also called the “modal” model (i.e., the model most frequently used), received enormous interest from the scientific community. At the same time, however, this model presented some limitations. For example, the model did not distinguish between modalities (e.g., verbal and spatial), and over the course of the years, it was replaced by other more detailed models.

Baddeley and Hitch (1974) introduced the concept of WM as a multi-storage mechanism. This model of WM proposed the existence of two slave systems (i.e., the phonological loop and the visuospatial sketchpad) and a central mechanism for actively maintaining and controlling the information (i.e., the central executive). Having three components, this model is generally known as the tripartite model. This model has been very influential and it can also predict several double dissociations in different disorders (e.g., lesions in the temporal parietal lobe provided evidence to support the existence of the phonological loop; patients with neglect presented patterns of performance that confirmed the presence of the visuospatial sketchpad; the patterns of performance of patients with frontal lobe damage and with Alzheimer’s disease confirmed the presence of the central executive component) (for an extensive discussion, see Baddeley 1992, 2003). Despite being very popular, this model also presented some problems, which led to subsequent refinements (Baddeley 2012).

One potential issue with the classical tripartite model is that the central executive component, which is a crucial part of the model, remains very elusive and difficult to objectively evaluate; for example, this component can only be measured indirectly, and this poses a series of methodological shortcomings (e.g., the existence of the central executive can be demonstrated using bi-factor models, but these models pose a series of statistical and methodological challenges) (for an extensive discussion, see Carretti et al. 2022). On the one hand, this led to the introduction of models (also known as modality-dependent models) distinguishing only between modalities (i.e., verbal and spatial) and not including the central executive component (Friedman and Miyake 2000). Conversely, other models posit that only tasks requiring a dual task, and in turn requiring higher degrees of attentional control, can be considered WM tasks. These models state that other tasks with only a single element that requires lower levels of attentional control can generally be considered short-term memory tasks (e.g., Engle et al. 1999). Since they do not present a distinction between modalities, they are usually known as modality independent. At the same time, other WM models have been proposed.

One alternative formulation of WM was proposed by Cornoldi and Vecchi (2003). This model encompasses a horizontal continuum distinguishing between modalities and a vertical continuum distinguishing between different levels of attentional control required. Since this model presents two dimensions (continuum), it is generally known as the “continua” model. This model shows some differences compared to other models. For example, this model assumes a differentiation between modalities in the horizontal continuum (e.g., distinguishing between simultaneous vs. sequential processes). At the same time, in this model, a clear distinction between STM and WM tasks on the vertical continuum is not present; tasks can simply require higher or lower levels of attentional control (Cornoldi and Vecchi 2000, 2003). Despite being less recognised, this model has been successfully tested with both typically and atypically developing children, showing that some children are specifically impaired in sequential vs. simultaneous tasks, and that it is often very hard to distinguish between STM and WM tasks since some tasks often require higher levels of attentional control (Carretti et al. 2022; Lanfranchi et al. 2004; Mammarella and Cornoldi 2005; Mammarella et al. 2006; Mammarella et al. 2018). The presence of several alternative WM models makes it necessary to statistically compare these models to determine which one is the most suitable.

Several authors comparing alternative models of WM favour the classical tripartite model (Alloway et al. 2006; Bayliss et al. 2003; Campos et al. 2013; Giofrè et al. 2013; Michalczyk et al. 2013). Gray and co-authors (Gray et al. 2017) found both evidence in favour of the tripartite model, but also for a model including a focus of attention factor (i.e., both models fitted the data well, making it very hard to compare them from a statistical point of view). Hornung et al. (2011) demonstrated that a bi-factor model provided a superior fit for the data. The bi-factor model is a model in which tasks load simultaneously on two factors, for example, a verbal task can load simultaneously on a verbal factor and on a common WM factor. It is worth mentioning, however, that this particular model, despite being widely used, also presents some limitations: (i) if only two indicators per factor are included, the loading of these indicators needs to be constrained to equality; (ii) factors are considered to be orthogonal (i.e., not correlated with each other), however, in several instances this is not the case; and (iii) this model allows us to precisely measure a common factor (e.g., a common WM factor), but at the same time other factors are considered to be residuals and it is difficult to understand the exact meaning and function of these residual factors (Gignac and Kretzschmar 2017). Recently, Carretti and co-authors (Carretti et al. 2022) found that the continua model presented several advantages and fitted the data well compared to several competing models. This latter study also used a bi-factor model, however, evidence in favour of the bi-factor model was not particularly strong.

### 1.2. Cross-Cultural Difference in WM

Evaluating the structure of WM in children from different countries is important since some differences might emerge between cultures. Roos et al. (2017) reviewed six papers on differences in WM in different countries. In their review the authors found that in three reports there were no differences in WM between African American vs. Caucasian American, bilingual Hispanic vs. Caucasian, and Chinese vs. American children (Buckhalt et al. 2007; Carlson and Meltzoff 2008; Lan et al. 2011). However, two studies did report some differences between Western and Asian cultures (Demetriou et al. 2005; Oh and Lewis 2008). In a study comparing Greek and Chinese children (age 7.5–15.1 years), Chinese children exhibited higher spatial and verbal WM with a particular advantage in spatial WM, even after accounting for processing speed differences. Similarly, a study comparing young British and Korean children found that Korean children exhibited higher spatial WM performances but similar performances in verbal WM tasks (Oh and Lewis 2008). Some evidence also indicates that Chinese preschoolers, as compared to American preschoolers, show comparable levels of storage capacity but have higher levels of attentional control and inhibition (Lan et al. 2011).

Advantages in WM in some countries might be related to the complexity of the Chinese writing system, which requires individuals to learn symbols through memorization. This emphasis on memorization is very common to many schools in East Asian cultures. Chinese children are, for example, required to master a logographic reading and writing system, which is considerably more complex as compared to any of the alphabetical systems used in Western countries. Children in China must learn 2570 characters during 6 years of primary school (Shu et al. 2003). This extensive training with complex characters may affect mental functioning all the way through from basic processing mechanisms to memory strategies and reasoning processes. The intensive character memorization emphasis is stressed in Japan as well where very young children are required to learn thousands of characters early in primary school (Muroya et al. 2017; Inoue et al. 2017). As mentioned above, while there is a host of research with adults, only a few studies have been performed with children. Japanese children, during the school years, receive intense training with complex characters that could potentially result in some differences in their WM capacity. However, the WM capacity of these children has rarely been evaluated, which makes research on this topic particularly timely.

### 1.3. The Relationship between WM and Intelligence

Intelligence can be broadly defined as the ability to reason, plan, solve problems, think abstractly, understand complex ideas, learn quickly, and learn from experience (Gottfredson 1997). Similarly to WM, several different models have been proposed for intelligence, however a distinction between fluid vs. crystallised intelligence has gathered a broader consensus (Horn and Cattell 1966). Some authors claim that intelligence and WM are hardly distinguishable (Colom et al. 2003; Kyllonen 2002). However, recent findings seem to confirm that intelligence and WM, despite being highly related and sharing about the 50% of the variance, are separable constructs, where about 50% of the variance is not shared (Conway et al. 2003; Engle et al. 1999; Giofrè et al. 2013). From a scientific perspective, however, it is important to understand which WM component is more closely related to intelligence.

There are several pieces of evidence indicating that WM and intelligence are closely related in children. Giofrè and co-authors (Giofrè et al. 2013), using the classical tripartite model, found that only WM and visuospatial-short term memory were related to intelligence. In a similar vein, Hornung and co-authors (Hornung et al. 2011), using a bi-factor model, found that the common factor explained a greater portion of variance in fluid intelligence, while other factors explained lower portions of the variance. Gray and colleagues (Gray et al. 2017) distinguished between fluid and visual intelligence and found that focus of attention explained a larger portion of the variance of both factors. These authors also presented a refined and reformulated WM model including a visuospatial WM factor, which was found to have a strong relationship with both visual and fluid intelligence. There is a lack of research with children, therefore, more research is needed to shed light on the structure of WM and its relationship with intelligence.

### 1.4. Aims and Rationale

The first aim of the current report was to test several alternative models for WM considering Japanese children. In particular, we tested: (i) a “modal” model only including a single WM factor (Atkinson and Shiffrin 1968); (ii) a modality-dependent model distinguishing between two factors (verbal vs. visuospatial) (Friedman and Miyake 2000); (iii) a modality-independent model, only presenting a distinction between a short-term memory (STM) and a WM factor (Engle et al. 1999); (iv) the classical tripartite model, distinguishing between two slave factors (i.e., visual and verbal STM) and a central executive factor (Baddeley and Hitch 1974); and (v) a bi-factor model, including a common WM factor, loading on all tasks, as well as a verbal and a visuospatial WM factor (Hornung et al. 2011). Once we established the structure of WM, we also aimed to evaluate the relationship between various WM factors, if more than one, and fluid and crystallized intelligence.

To sum up, the main aim of the current report was to test several competing models of WM to establish the most suitable model in Japanese children. Once we have established the best fitting model, we also aimed to investigate the relationship between various WM components and fluid and crystallized intelligence.

## 2. Materials and Methods

### 2.1. Participants

The sample initially included 173 children. Some children (n = 3) were absent during one of the administrations and were excluded from the final sample. The final sample included a total of 170 children (104 male and 66 female, 96 second and 74 fifth graders; M_age_ = 112.84 months, SD = 18.04). Children were recruited through local mainstream primary school programs in Japan. A criterion for inclusion was the use of Japanese as a first language.

### 2.2. Measures

#### 2.2.1. Working Memory

WM tasks were selected from a battery of tasks used in several other studies with good psychometric properties (Allen and Giofrè 2021; Allen et al. 2020; Donolato et al. 2019; Giofrè et al. 2014; Giofrè et al. 2018). Verbal tasks required an adaptation to Japanese language which was done by a bilingual speaker. The partial credit score (the number of correct objects identified in the right order) was used (Giofrè and Mammarella 2014). This method allows us to obtain information from partially recalled series, for example, using a traditional span procedure, if the subject is able to identify seven objects from a list of eight objects, the corresponding performance is zero, while the partial credit score allows us to obtain some information from partially recalled lists (i.e., seven out of eight items are correctly recalled). This method was adopted with adults, showing that the reliability increased substantially using this procedure (Unsworth and Engle 2006, 2007). This procedure, which stresses the importance of information obtained with the most difficult (longest) lists of items to recall, also emphasises the role of STM tasks in explaining intelligence (Unsworth and Engle 2006, 2007). For example, Giofrè and Mammarella (Giofrè and Mammarella 2014) found that this procedure provided higher reliability estimates, while at the same time also increased the predictive power of STM and WM tasks for intelligence.

##### Verbal

Three measures of verbal WM were administered: the number span task (NST), the word span task (WST), and the listening span task (LST).

*The number and word span tasks* required children to repeat lists of auditorily presented numbers (Cronbach’s *α* = .89) or words (*α* = .88) that they had heard in forward order. Two trials for each span length, starting from a length of two to a length of eight words/numbers, were presented. The reliability, calculated on the current sample, was relatively high for both numbers (*α* = .89) and words (*α* = .88).

*In the listening span task (LST)* (Daneman and Carpenter 1980), children listened to sets of sentences from two to five sentences. The first set contained two sentences, and the number of sentences increased gradually in subsequent sets. After hearing each sentence, children were asked to evaluate whether the sentence was true or false. After each set of sentences, children were asked to recall the first word of each sentence in the same order as they were presented. Two trials per span length were presented. The reliability, calculated on the current sample, was relatively high (*α* = .90).

The Japanese version of the word span and listening span tasks were designed with nouns that meet three criteria: two characters, two syllables, and two morae (a phonological unit of a language), which were selected from the Textbook Vocabulary Corpus (Tanaka et al. 2011). These nouns are commonly used in approved textbooks for lower primary school children. In designing these tasks, we took care to ensure that the vowel combinations of the words within the same trial sequence did not overlap. For example, the Japanese words ‘uma’ (うま) and ‘kusa’ (くさ) have the same vowel combination (‘u’ and ‘a’), while ‘neko’ (ねこ) has a different vowel combination (‘e’ and ‘o’). To avoid repetition and the phonological similarity effect, we ensured that words with identical vowel combinations were not presented in the same trial sequence. Furthermore, each to-be-remembered item (word) appeared only once in the WM task. Additionally, in the listening span task, we employed three-word sentences adhering to the Subject-Object-Verb (SOV) structure as stimuli. Examples of these sentences include “Ears eat rice” (みみで ごはんを たべる), which is an incorrect version of “Mouth eats rice” (くちで ごはんを たべる), and “Bears live in the mountains” (くまは やまに すんでいる). Upon hearing the sentence read aloud, participants were required to evaluate whether the content of the sentence was semantically coherent while simultaneously recalling and memorizing the first word of each sentence, i.e., ‘mimi’ (みみ) and ‘kuma’ (くま).

##### Spatial

Three measures of spatial WM were administered: the matrices span task (MST), the Corsi block task (CBT), and the dot matrix task (DMT).

*The matrices span and Corsi block tasks* required participants to memorize and recall the positions of cells that appeared briefly (for 1 s) in different positions on the screen (on a 5 × 5 grid in the matrices span task, *α* = .91, and on 9 blocks displayed on the screen in the Corsi block task, *α* = .90). After a series of cells had been presented, the children used a mouse to click on the locations where they had seen a cell appear in the order in which the cells were presented. Two trials for each span length, from two to eight blocks/matrices were presented. The reliability, calculated on the current sample, was relatively high for both matrices (*α* = .91) and Corsi block (*α* = .90) tasks.

*In the dot matrix task* (Miyake et al. 2001), children were presented with sets of matrix equations (i.e., addition with lines in a grid of dots). After each equation, a dot appeared in a 5 × 5 grid, and children were asked to remember its position, so they had to verify the matrix equation while simultaneously remembering the dot’s location. The sets of matrices were presented in four series of increasing length from two to five dots. Participants then had to recall the locations of the dots in the correct order. Two trials for each span length, from two to five dots, were presented. The reliability, calculated on the current sample, was relatively high (*α* = .90).

#### 2.2.2. Intelligence

##### Fluid Intelligence

*The Japanese version of the Cattell Culture Fair Intelligence Test Scale 2* (Cattell and Cattell 1960): The test consists of two forms, A and B. Each form includes four timed subtests of nonverbal fluid reasoning (series, classifications, matrices, and topology) with items of increasing difficulty within each subtest. We calculated two scores from the sum of correct answers for the form A and form B separately (reliability test–retest, *r* = .84).

##### Crystallized Intelligence

Two tasks from the Japanese adaptation of the WISC-IV (Wechsler and Japanese WISC-IV Publication Committee 2010) were presented. Vocabulary typically requires providing definition of words presented auditorily (reliability test–retest, *r* = .80).

Similarity typically requires evaluating two words and explaining why they are similar to each other (e.g., a poet and a painter are both artists) (reliability test–retest, *r* = .85). Both tasks present progressively difficult items and tasks are discontinued if a particular child fails to respond to more difficult items and receives 5 consecutive scores of 0 points.

### 2.3. Design & Procedure

The six WM measures were administered in a randomized order. For the WM tasks, all stimuli were presented on a 15.6-inch monitor with a viewing distance of 50 cm; all children were individually tested in quiet rooms at their schools. Stimulus presentation was controlled by E-PRIME software (version 2.0, Psychology Software Tools, Sharpsburg, PA, USA). Cattell intelligence tests were collectively administered in the classroom, while tasks from the WISC-IV were individually administered.

### 2.4. Data Analytic Approach

The R program (R Core Team 2022) with the “lavaan” library (Rosseel 2012) was used. Model fit was assessed using indices and criteria suggested by Hu and Bentler (1998): the chi-square (*χ*^2^), the comparative fit index (*CFI*), the non-normed fit index (*NNFI*), the standardized root mean square residual (*SRMR*), and the root mean square error of approximation (*RMSEA*). The Akaike information criterion, AIC, was used for the model comparison of non-nested models, while the chi-square difference test (Δ*χ*^2^) was used for nested models.

Variance partitioning analyses were performed using the latent correlation matrix. The correlation matrix was used for calculating the R^2^ using the “mat.regress” function available in the “psych” package (see Allen and Giofrè 2021 for a similar procedure).

## 3. Results

### 3.1. Preliminary Analyses

Grade was partialled out of all analyses to remove its influence on the data (for a similar procedure, see Giofrè et al. 2018; Donolato et al. 2019). Partialling out for grade was necessary to increase the statistical power of our analyses. However, additional analyses were performed to check the appropriateness of this procedure (see additional analyses). Correlations, both raw and covaried, as well as descriptive statistics, are presented in Table 1.

### 3.2. Confirmatory Factor Analysis (CFA)

#### 3.2.1. Working Memory

Several alternative WM models were tested to ascertain the structure of WM in Japanese children: in Model 1 we tested a single factor model; in Model 2 we tested a model distinguishing between a verbal and a spatial factor (modality dependent); in Model 3 we tested a model distinguishing between WM and STM factors (modality independent); in model 4 we tested a model distinguishing between short term memory verbal (STM-V) and visuospatial (STM-VS) factors and a WM factor (tripartite model); in model 5 we tested a model presenting two WM factors (verbal and visuospatial), but also a common WM factor loading on all tasks (bi-factor model). The fit of each model is presented in Table 2.

**Figure 1:**
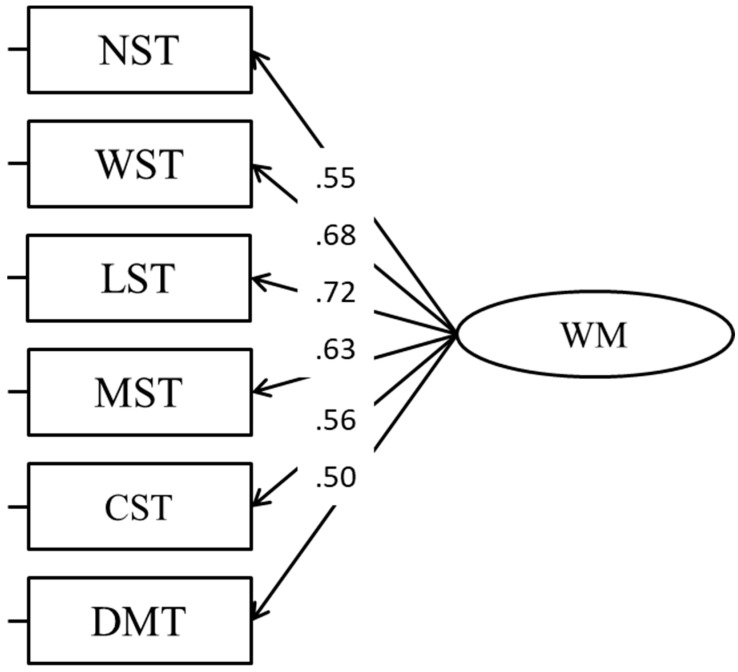
A single WM factor (“modal” model) loading on all tasks.

As a “modal” model (CFA, Model 1; Figure 1), we tested a single WM factor (Atkinson and Shiffrin 1968). However, the fit of the model was not satisfactory (Table 2), so we decided to test alternative models.

**Figure 2:**
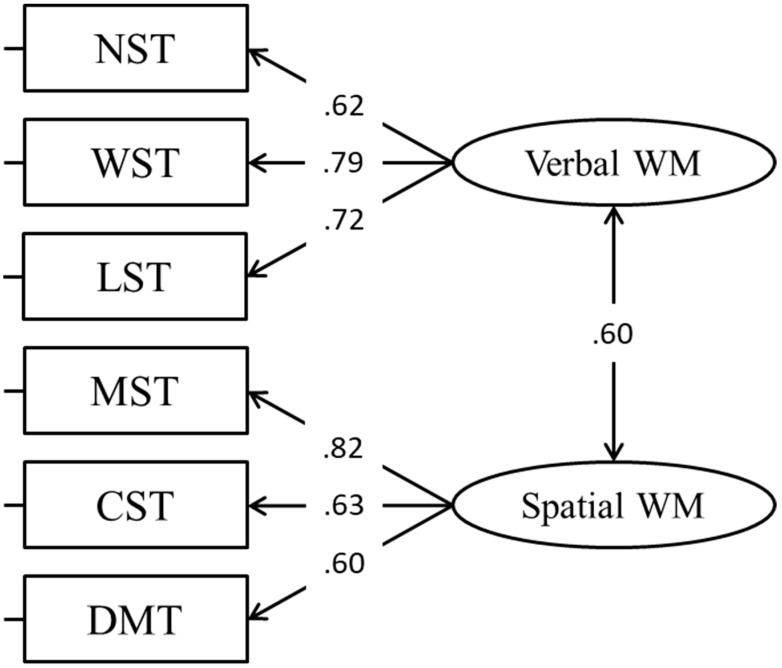
Modality-dependent model.

In this modality-dependent model (CFA, Model 2; Figure 2), WM was considered to have two factors, namely, verbal and spatial (Friedman and Miyake 2000). The fit of the model was considerably better (Table 2), however, we decided to test other alternative models to see whether they provided a better fit to the data.

**Figure 3:**
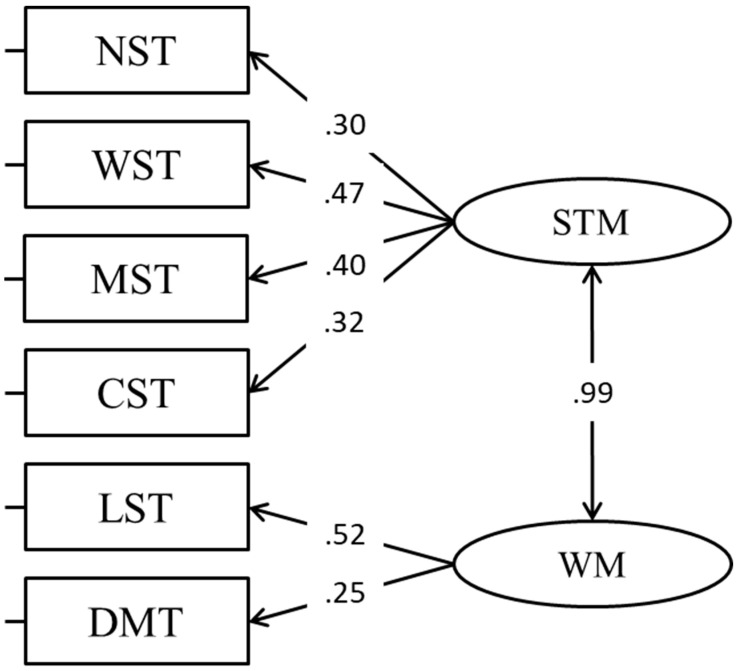
Modality-independent model.

In a subsequent model, a modality-independent model (distinguishing between STM and WM) was fitted (Engle et al. 1999). In this model, the correlation between the two factors was extremely high, making them empirically undistinguishable (Figure 3). In addition, the fit of the model was poor (CFA, Model 3; Table 2). For these reasons, this model was not retained.

**Figure 4:**
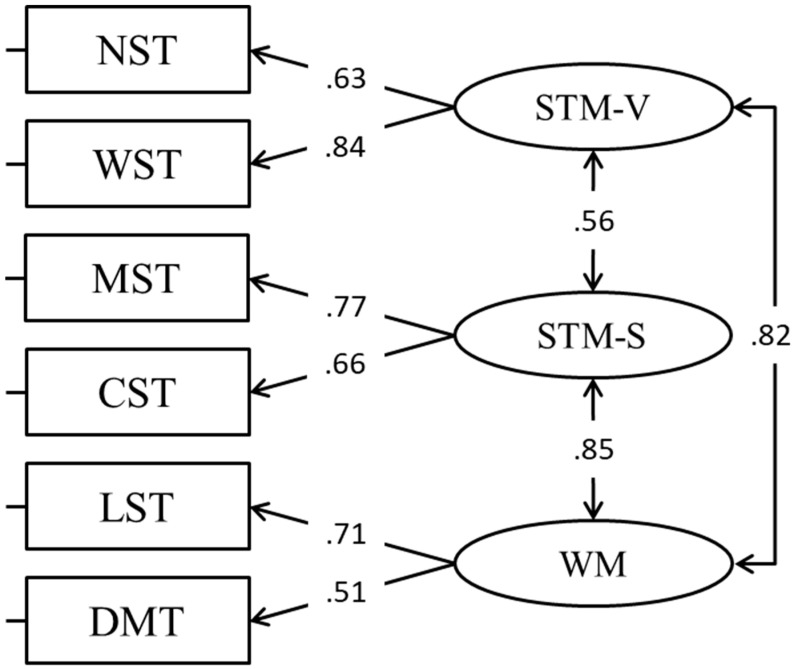
The tripartite WM model.

We also tested the classical tripartite WM model (CFA, Model 4) considering short term memory verbal (STM-V), short-term memory spatial (STM-S), and a WM factor (Baddeley and Hitch 1974). In this model, correlations between the short term memory verbal and spatial factors with the WM factor were very high (Figure 4), making these factors hardly separable. Also, this model as compared to previous models (Table 2) did not provide a better fit and so was not retained.

**Figure 5:**
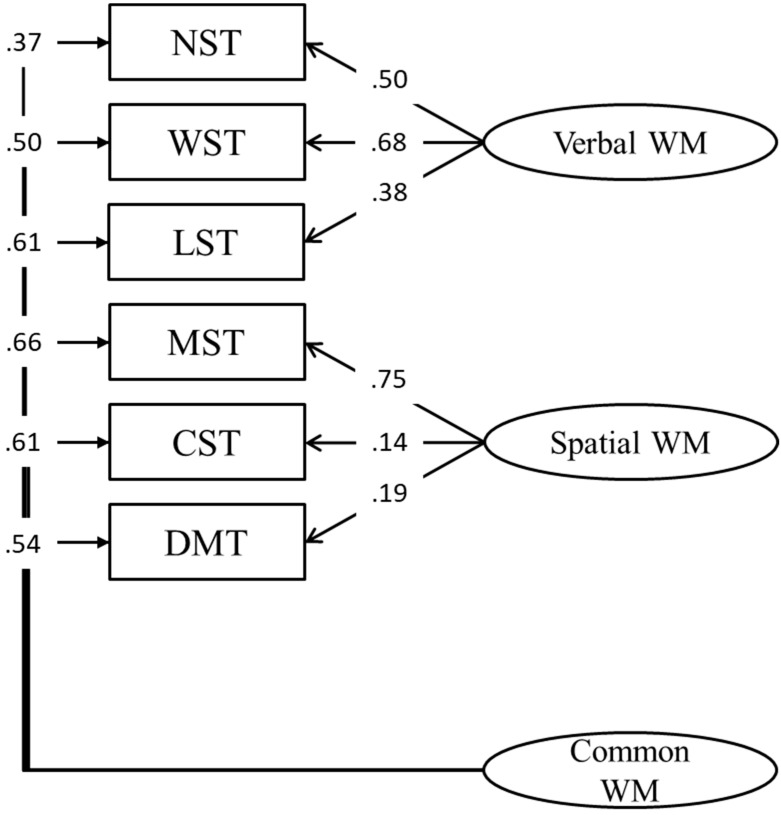
Bi-factor model including verbal, spatial, and common WM factors.

In a bi-factor model, we tested three orthogonal factors: verbal and spatial WM, loading on verbal and spatial tasks respectively, and a common WM factor loading on all variables (CFA, Model 5; Figure 5). The initial model did not converge due to a problem with negative variance, so for this reason the variance of the matrix span test (MST) was set to zero. This model provided a good fit to the data (Table 2), showing lower RMSEA and SRMR and higher CFI and NNFI. However, it also presented with a higher AIC as compared to model 2. The AIC generally penalizes more complex models over models with fewer parameters to be estimated, therefore, despite the model having a very good fit, we decided to retain model 2 for further analyses.

#### 3.2.2. Working Memory and Intelligence

**Figure 6:**
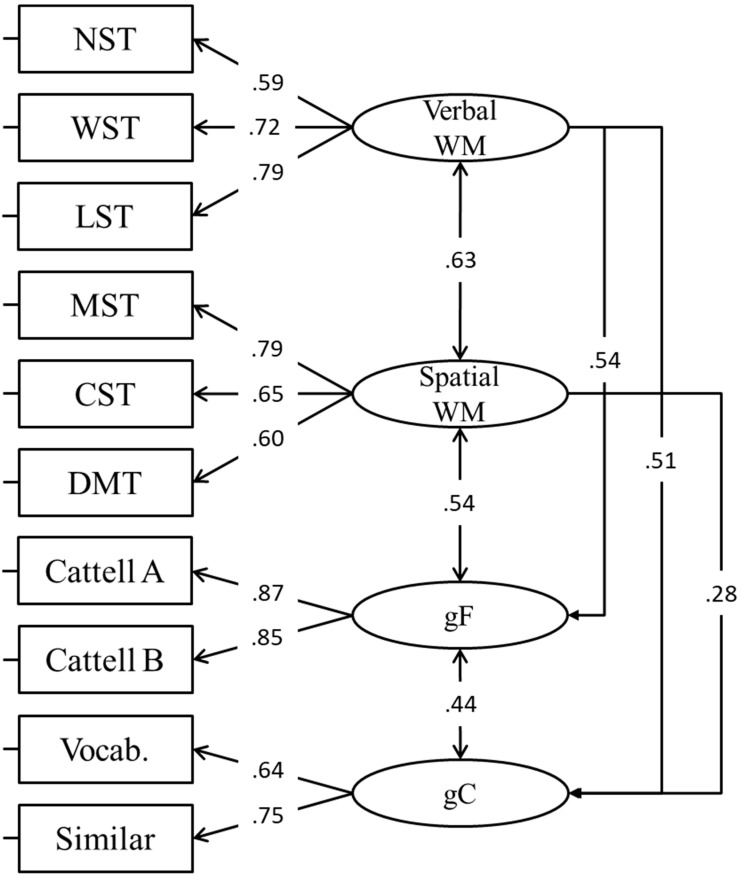
SEM model in which the g factor is predicted by WM factors. Structural model shown for clarity. Dashed lines are not statistically significant. gF = fluid intelligence; gC = crystallized intelligence.

In a subsequent model, intelligence (both fluid and crystallized) was incorporated alongside WM (CFA, Model 6). This measurement model, which should not be compared with previous CFA models (as it also includes intelligence measures), was needed to (i) understand the relationship between verbal and spatial WM factors with fluid and crystallized intelligence and (ii) provide the correlation matrix needed for subsequent analyses. The model provided a good fit with the data (Table 2) and results are presented in Figure 6.

### 3.3. Variance Partitioning

**Figure 7:**
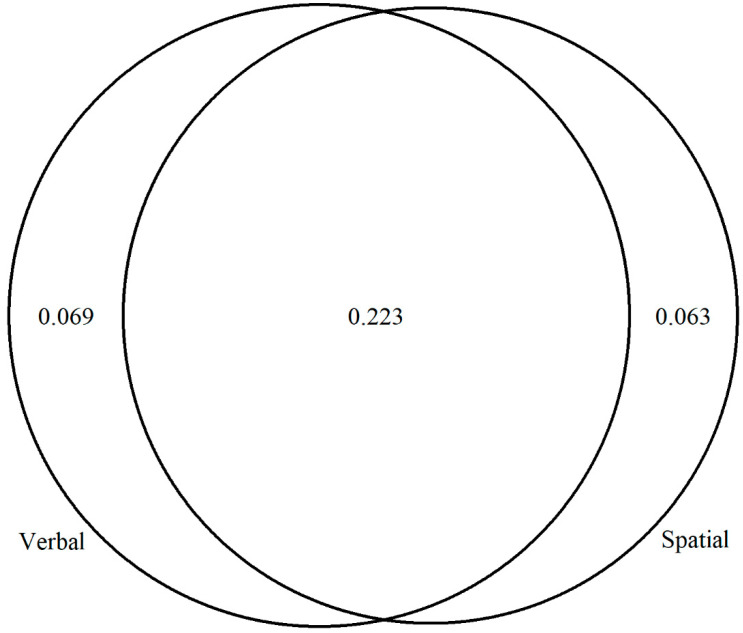
Variance explained for the fluid intelligence factor by the verbal and spatial WM factors. The shared area represents the joint contribution, while the non-shared area represents the unique contribution of each WM factor. Verbal = verbal WM; Spatial = spatial WM.

The correlation matrix from the last CFA model (i.e., Model 6) was used in subsequent analyses to understand the role of each individual WM factor as well as their joint effect on both fluid and crystallized intelligence. Concerning fluid intelligence, the joint contribution of verbal and spatial WM explained the largest portion of the variance. In addition, both verbal and spatial WM explained a unique portion of the variance of fluid intelligence (Figure 7).

**Figure 8:**
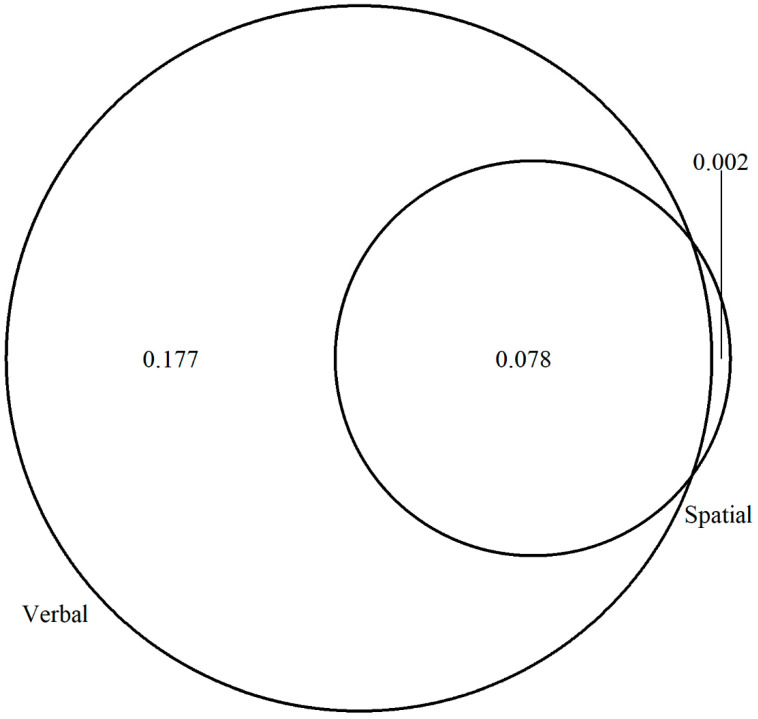
Variance explained for the crystallized intelligence factor by the verbal and spatial WM factors. The shared area represents the joint contribution, while the non-shared area represents the unique contribution of each WM factor. Verbal = verbal WM; Spatial = spatial WM.

As for the crystallized intelligence factor, the unique contribution of verbal WM explained the largest portion of the variance, while the shared contribution of both verbal and spatial WM explained a smaller portion. Interestingly, the unique variance accounted for by the spatial WM factor was a very small portion, which was close to zero (Figure 8).

### 3.4. Additional Analyses

To maximize the statistical power in the analyses above, we used the entire sample partialling out the effect of grade. However, in a subsequent set of analyses, we wanted to test whether the same factorial structure was tenable in the two age groups: younger children (from 7 years and 5 months to 8 years and 9 months) and older children (from 9 years and 9 months to 11 years and 6 months). For this reason, we decided to perform some exploratory multigroup confirmatory factor analyses (MG-CFA). In the first model, the factorial structure (CFA, Model 6) was tested in the two groups. Results demonstrated a good fit of the data (Table 2), showing that the same structure was tenable between the two groups (MG-CFA, Model 1). We then tested the loading invariance in the two groups (MG-CFA, Model 2). The chi-square difference test was not statistically significant, meaning that loadings could be constrained in the two groups, Δ*χ*^2^(6) = 7.28, *p* = .296. We then constrained the residual variance (MG-CFA, Model 3), which was also not statistically significant in this case, Δ*χ*^2^(10) = 18.08, *p* = .054. Results indicate that the two age groups presented a similar pattern of relationships, meaning that they could be considered together in the analyses.

## 4. Discussion

In the current report, we aimed to compare several alternative WM models in a sample of Japanese children. We found that a model distinguishing between modalities presented a very good fit and was superior in terms of AIC compared to the others. It is worth mentioning, however, that we found some evidence for the presence of a common WM factor using the bi-factor model. This bi-factor model also had a very good fit but was somewhat less parsimonious (i.e., had a higher AIC and fewer degrees of freedom). This reflects a general problem with the bi-factor model, which needs a number of additional parameters to be estimated. Also, this model has strong assumptions, for example, the common (general) factor is extracted first while the other factors are somewhat residual (but see Gignac 2008 for a discussion). The results are therefore also compatible with a higher-order model including a common WM factor at the top of the hierarchy. However, estimating this model imposes an equality constraint, making it statistically equivalent to the two factor models, meaning that is impossible to test the difference between the two (Gignac 2016; Zinbarg et al. 2007). For all these reasons, despite the model with a common WM factor (bi-factor or hierarchical) being statistically plausible, we decided to select a more parsimonious model only distinguishing between two factors (verbal and visuospatial WM) for further scrutiny. Since we were also interested in evaluating the shared contribution of these factors, we decided to use variance partitioning. This allowed us to estimate the variance explained by the joint contribution of these two WM factors as well as portions of unique variance independently explained by each factor.

Variance partitioning analyses revealed that the joint contribution of verbal and spatial WM explained the largest portion of the variance in fluid intelligence, with some residual variance explained by the unique variance of both verbal and spatial WM. Such a result is compatible with previous research indicating that WM, independent of the modality, explains a very large portion of the variance of fluid intelligence (Giofrè et al. 2013, 2017; Hornung et al. 2011). Concerning crystallized intelligence, we found that the verbal WM factor explained the largest portion of the variance, with the shared contribution between verbal and spatial WM factors explaining a residual portion of the variance. This result is very important since it seems to indicate that verbal and spatial WM factors are linked to different intelligence components. It is reasonable to conclude that verbal WM is particularly involved in tasks in which a phonological elaboration of the stimulus is needed (e.g., Haavisto and Lehto 2005). In a similar vein, fluid intelligence requires attentional control to a larger extent, therefore, it is not surprising that the shared variance between the verbal and spatial WM factor explains the largest portion of the variance (e.g., Engle et al. 1999). It is also worth mentioning that to solve fluid intelligence tasks, children need to be adaptable and to draw on several different resources rather than mainly recalling facts. Also, fluid intelligence tasks are generally based around visual elements, which also heavily involve visual processing, while at the same time reasoning abilities are highly taxing on verbal processing. Finally, this pattern of results is very similar to results by Kane and colleagues in a sample of adults, confirming that verbal tasks are somewhat more related to crystallized intelligence while spatial tasks are more related to fluid intelligence (Kane et al. 2004).

From a theoretical perspective, we can conclude that a distinction between modalities is very robust and tenable. This result is in line with the recent report by Carretti and co-authors (Carretti et al. 2022) finding that modalities are distinguishable within WM. As for the presence of a common WM factor, we did not find conclusive evidence for the bi-factor model, which fitted the results well, but it was somewhat more difficult to estimate, for example, we were forced to fix some residual variance, which is not ideal. As for a hierarchical model (i.e., a model with a superordinate factor at the top), this is also plausible but statistically indistinguishable from a modality dependent (verbal vs. spatial) two-factor model and requires equality constraints to be fitted, which is also not ideal (see Gignac 2016 on this point). For all these reasons, our results seem to be compatible with those of Carretti and co-authors (Carretti et al. 2022). We cannot disprove the presence of a common WM factor, but this factor is elusive and very hard to estimate from a statistical point of view.

It is worth noting that the procedure we used (i.e., the partial credit score) can partly explain this pattern of results. When using the partial credit score, tasks that typically tap STM also tap central executive resources. This is possible because the partial credit score extracts information that would not be extracted using a traditional scoring procedure (e.g., the simple span). Unsworth and Engle (2006, 2007), for example, demonstrated that STM and WM tasks perform similarly and present similar predictive power on intelligence when using the partial credit score. Other research also found that spatial tasks are somewhat special, as their relationship with fluid intelligence seems to be higher as compared to verbal STM tasks, which might indicate that, in general, spatial WM tasks require higher levels of attentional control as compared to verbal tasks (Giofrè et al. 2013; Kane et al. 2004).

The current report also has some important practical and clinical implications. Firstly, the results show a distinction between modalities in WM, which could be leveraged in an educational setting. For example, Giofrè and co-authors (Giofrè et al. 2018) found that verbal and spatial WM are probably separable and contribute differently to the prediction of mathematical achievement. In a similar vein, Allen and Giofrè (2021) found that different mathematical achievement tasks might require spatial and verbal WM to different extents, with tasks such as geometry requiring spatial WM to a larger extent and other tasks such as algebra being largely influenced by verbal WM. It is also likely that verbal and spatial WM abilities are differentially involved in mathematical achievement during the course of development, with spatial WM being more important in very early stages and verbal WM becoming more important later on (Allen et al. 2021). It is also worth mentioning that research has shown that the WM–mathematics relationship changes dependent on age and mathematical sub-component (Zhang et al. 2022; Gordon et al. 2022). A modality distinction can also be important in clinical settings, with children with different disabilities being selectively impaired on tasks depending on which modality is affected (Mammarella et al. 2018). In addition, Japanese data seem to be similar to those obtained in other countries (e.g., Italy or the UK). For all these reasons, it seems reasonable to maintain a modality distinction within WM because it is useful from a clinical and practical perspective.

The results of the present study need to be replicated in future studies. One of the limitations of the present report is that we only had six WM tasks, making it hard to test more articulated models (e.g., models distinguishing between spatial simultaneous and spatial sequential processes (Cornoldi and Vecchi 2003)). It would also have been very interesting to include other measures, such as updating and binding tasks (see, for example, Gray et al. 2017) or processing speed tasks, which can predict a portion of variance in intelligence over and above the variance predicted by WM (Gordon et al. 2020). Regrettably, our agreement with the schools only allowed us to test children for a limited amount of time, thus a larger WM assessment would have been unfeasible under these circumstances. It would also be important to test younger and older children as well—or even better—to take a longitudinal approach, which would allow us to generalize the results even further. Here again this was not possible due to practical reasons (e.g., longitudinal projects tend to be very demanding in terms of resources).

## 5. Conclusions

Despite the aforementioned limitations, we believe that the current report testing a large number of Japanese children has important implications in several areas (theoretical, practical, and clinical), since the research on this topic is scarce and needs a much closer investigation in future literature.

## Figures and Tables

**Table 1:** Correlations among WM and intelligence measures.

	1	2	3	4	5	6	7	8	9	10
1. NST	―	0.61	0.55	0.40	0.39	0.33	0.35	0.35	0.41	0.40
2. WST	0.53	―	0.66	0.51	0.43	0.38	0.37	0.38	0.42	0.43
3. LST	0.41	0.56	―	0.56	0.54	0.56	0.53	0.52	0.57	0.59
4. MST	0.25	0.38	0.38	―	0.62	0.63	0.45	0.45	0.41	0.44
5. CST	0.25	0.29	0.37	0.51	―	0.51	0.46	0.43	0.40	0.40
6. DMT	0.15	0.20	0.36	0.50	0.36	―	0.41	0.38	0.37	0.44
7. Cattel A	0.23	0.25	0.41	0.33	0.34	0.27	―	0.76	0.41	0.49
8. Cattell B	0.26	0.29	0.44	0.36	0.34	0.27	0.74	―	0.31	0.39
9. Vocabulary	0.21	0.22	0.31	0.14	0.15	0.06	0.24	0.16	―	0.70
10. Similarities	0.19	0.21	0.33	0.16	0.14	0.14	0.35	0.26	0.48	―
M	32.15	26.39	12.36	43.92	40.79	14.56	26.79	27.16	20.41	15.82
SD	5.74	8.09	6.09	14.25	10.5	6.07	6.22	6.61	7.43	5.55
Skew	−0.42	0.62	0.15	−0.06	−0.32	−0.04	−0.27	−0.59	0.90	0.34
Kurtosis	−0.55	0.82	−0.73	−0.16	−0.15	−0.96	−0.18	−0.14	0.49	−0.73

*Note*. Correlations covaring for age below the diagonal and raw correlations above the diagonal. NST = number span task, WST = word span task, LST = listening span task, MST = matrix span task, CST = Corsi span task, DMT = dot matrix task. Correlations higher than 0.15 are statistically significant at *p* < .05.

**Table 2:** Model fit statistics.

	*χ* ^2^	*df*	*p*	*RMSEA*	*SRMR*	*CFI*	*NNFI*	*AIC*
CFA								
WM								
Model 1	56.84	9	0.000	0.177	0.086	0.819	0.699	6643
Model 2	13.41	8	0.099	0.063	0.043	0.980	0.962	6601
Model 3	56.84	8	0.000	0.190	0.086	0.815	0.654	6645
Model 4	28.87	6	0.000	0.150	0.063	0.914	0.784	6621
Model 5	6.51	4	0.164	0.061	0.023	0.991	0.964	6603
WM & g								
Model 6	33.16	29	0.271	0.029	0.039	0.992	0.987	10,592
MG-CFAs								
Model 1	24.27	16	0.084	0.078	0.050	0.969	0.942	6633
Model 2	29.95	20	0.071	0.077	0.071	0.963	0.944	6631
Model 3	36.58	26	0.081	0.069	0.089	0.961	0.954	6625

*Note*. The first five models only include WM tasks. Model 6 also includes intelligence measures. Multigroup models were performed between grades. CFA = confirmatory factor analyses; MG-CFA = multigroup CFA.

## Data Availability

The participants of this study did not give written consent for their data to be shared publicly, so due to the sensitive nature of the research, supporting data is not available.
